# The positive implication of natural antioxidants on oxidative stress-mediated diabetes mellitus complications

**DOI:** 10.1016/j.jgeb.2024.100424

**Published:** 2024-09-10

**Authors:** Shouvik Mallik, Bijoy Paria, Sayed Mohammad Firdous, Hesham S. Ghazzawy, Nashi K. Alqahtani, Yong He, Xiaoli Li, Mostafa M. Gouda

**Affiliations:** a Department of Pharmacology, Calcutta Institute of Pharmaceutical Technology & AHS, Uluberia, Howrah, West Bengal, India; b Date Palm Research Center of Excellence, King Faisal University, Al Ahsa, Saudi Arabia; c College of Biosystems Engineering and Food Science, Zhejiang University, Hangzhou 310058, China; d Department of Nutrition & Food Science, National Research Centre, Dokki, Giza 12622, Egypt; e Central Laboratory for Date Palm Research and Development, Agriculture Research Center, Giza 12511, Egypt

**Keywords:** Diabetes mellitus (DM), oxidative stress (OS), Antioxidants, Insulin resistance

## Abstract

The complementary intervention to modulate diabetes mellitus (DM) metabolism has recently brought the global attention, since DM has become among the global burden diseases. Where, several related pathways elevate the production of superoxide in consequences. For example, the flux of glycation-derived end products (AGEs) could lead to the deactivation of insulin signaling pathways. In that context, many vitamins and phytochemicals in natural sources have high antioxidant impacts that reduce oxidative stress and cell damages. These chemicals could be applied as bioactive antidiabetic agents. Their mode of actions could be from regulating the intracellular reactive oxygen species (ROS) which cause several pro-inflammatory pathways related to the oxidative stress (OS) and DM. Besides, they have a great potential to control the epigenetic mutations and hyperglycemia and help in back the blood glucose to the normal level. Therefore, the current review addresses the important role of natural functional antioxidants in DM management and its association with its OS complications.

## 1 Introduction

**Fig. 1:**
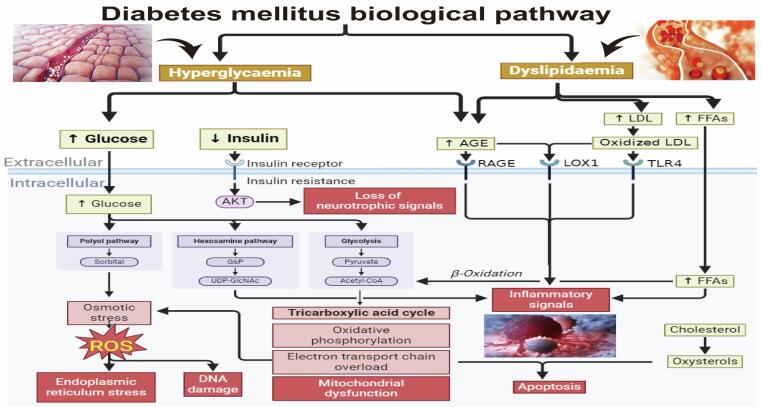
Diabetes mellitus (DM) metabolic mechanism of action and its relation to cardiovascular diseases**.**

The condition of diabetes mellitus (DM) is described as a part or total deficiency in insulin action, specifically pathway-specific resistance to insulin and progression of specific illness in the glomerulus, the retina peripheral nerve, which defines all forms of diabetes. A variety of metabolic disorders result in DM which is defined as chronic hyperglycemia that causes disruption in insulin formation, function, or both.^1^ Where, it is associated with the increase in the incidence of atherosclerotic disease which impacts the arteries that connect the brain, heart that lead to cardiomyopathy as a consequence associated with cardiovascular risks (three to eight-fold), because of impaired glucose tolerance and diabetes (Fig. 1). Therefore, over 30 % of patients who have been admitted to the hospital and suffer from acute myocardial infarction suffer from diabetes, and close to 35 % suffer from diminished glucose tolerance.^2^ At present, several countries in the world are experiencing a worldwide diabetes “epidemic” that is quickly spreading throughout the globe.^3^ Diabetes-related chronic hyperglycemia is a sign of organ and tissue dysfunction, damage, and even failure, the emergence of microvascular (retinopathy and nephropathy) as well as macrovascular (cardiovascular conditions) problems..^4^

As presented in Fig. 1 that among the DM important metabolites related to DM are the ROSs that are products of oxygen metabolism and that are produced by the cellular mitochondria.^5^ These molecules including superoxide and hydroperoxyl radicals, singlet radicals, hydroxyl radical peroxynitrite, and nitric oxide.^6^ Altogether, the most common ROS kinds are superoxide and hydroxyl radicals, peroxynitrite and nitric Oxide, singlet radicals, hydroperoxyl radicals.^6^ Altogether, these mentioned different types has specific mechanism of action for their distinct characteristics and neutralization methods.^6^ In depth, the superoxide anion (O_2_^–^), formed when molecular oxygen gains an extra electron, is a moderately reactive molecule and a precursor to other ROS. Superoxide dismutase (SOD) neutralizes it by converting it into hydrogen peroxide (H₂O₂). Additionally, hydrogen peroxide, which consists of two hydrogen atoms bonded to an oxygen–oxygen single bond, is less reactive, can diffuse across membranes, and may lead to the formation of more reactive species. These actions could be regulated enzymatically by Superoxide dismutase (SOD), glutathione peroxidase (GPx), glutathione reductase (GR), catalase, paraoxanase (PON), and other antioxidant enzymes exist that could neutralize H₂O₂ by converting it to water (H₂O) and oxygen (O₂)..^8^

On the other hand, these molecules are extensively involved in the normal intracellular signaling and cellular regulations, including apoptosis induction, and the immune responses.^6^ Therefore, the antioxidant defense (AOD) system defends the biosystem by regulating the ROS' negative consequences following the enzymatic and non-enzymatic molecular stimulations. Non-enzymatic antioxidant resistance such as reduced glutathione (GSH), melatonin, tocopherols, carotenoids, phycocyanin, polyphenols, ceruloplasmin, retinol, ascorbate carnosine.^9^; where they could effectively maintain ROS to the normal levels in the human cells.^9^, ^10^ Under several pathological circumstances, including diabetes, the redox equilibrium can be disrupted, resulting in detrimental effects into the stressed cells..^6^

As an important example, the physiological non-enzymatic defense against hydroxyl radical (•OH), which is highly reactive and damaging to biomolecules, could be effectively neutralized by the direct scavenging of Vitamin C and Vitamin E.^7^ Moreover, carotenoids and Vitamin E showed an effective regulatory implications on the singlet oxygen (^1^O₂), which is an excited form of oxygen with electrons in a higher energy state, highly reactive, and capable of causing significant cellular damage.^10^ Additionally, peroxynitrite (ONOO^–^) is produced by the reaction of nitric oxide (NO) with superoxide, is highly reactive, and can cause nitration of proteins and lipids, and is reduced by peroxiredoxins and glutathione..^8^

Indeed, there is still a research gap in how the plant-derived antioxidants can play an important role with their antioxidative effect against the hyperglycemia OS-mediated diabetic complications, specially in cardiovascular disease, retinopathy, and nephropathy according to their effective mode of actions. Being naturally derived products, there are several proves to be a cost-effective alternative to the most synthetic antidiabetic medications out on the market. Thus, the current review discusses the potential role of natural antioxidants from different sources such as sweet potato, broccoli, almonds, legumes, brussels sprouts, and other plant sources for the complementary treatment of diabetic complications.

## 2 Effects of hyperglycemia on microvascular complications and its molecular mechanism with oxidative stress development along with DM

The prolonged exposure to the high glucose levels causes microvascular problems in diabetics that are associated with the severity of diabetic-related tissue damage, like atherosclerosis, dyslipidemia, and hypertension.^11^ The connection between hyperglycemia ROS and diabetic complications selectivity in cellular sensitivity remains a complicated issue that researchers are working to understand. Whereas, hyperglycemia generalized targets specific cell types because these cells do not down-regulate glucose absorption as extracellular glucose levels increase.^12^ When there is an increase in glucose levels the vascular endothelial cells do not show a significant change in their glucose transport rates, which results in the presence of intracellular hyperglycemia..^13^

**Fig. 2:**
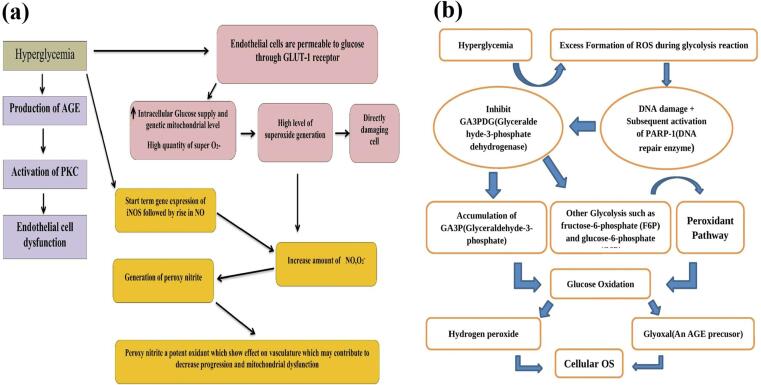
**(a)** Hyperglycemia induce os and vascular complication and disease progression**. (b)** Molecular mechanisms of oxidative stress development in diabetes mellitus**.**

Indeed, microvascular disease is more common in tissues that have insulin-independent glucose absorption, like kidneys retinas as well as vascular endothelium. Through altering blood flow, the permeability of endothelial cells, and extravascular proteins are degraded and then coagulated and coagulation, these metabolic damages create organ malfunction. Recent research shows a definite link between high blood pressure (BP) and the progression of nephropathy and retinopathy.^14^, ^15^ In addition, contemporary research provides compelling evidence of the direct association between BP levels and the development of nephropathy as shown in Fig. 2**a**. Where, superoxide ROS directly harms cells and triggers four main causes of diabetic problems. These problems include the increase in endothelial glucose levels by GlUT1 receptor, AGE production, peroxynitrite and iNOS gene expression that causes harmful implications on the cells and their mitochondrial dysfunction..^16^

**Fig. 3:**
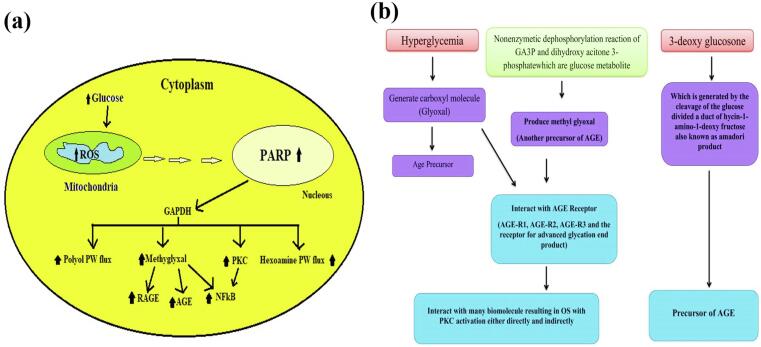
**(a)** Schematic showing elements of the unifying mechanism of hyperglycemia-induced cellular damage. **(b)** Various precursor of AGE.

Under the conditions of hyperglycemia glycolysis, an increase in the production of ROS occurs that causes DNA damage and the activation of poly-ADP-ribose Polymerase 1 (PARP1) which is an enzyme that repairs DNA.^17^ Whereas. it inhibits GA3PDG (Glyceraldehyde-3-phosphate dehydrogenase), and it leads to other glycolysis such as fructose-6-phosphate and glucose-6-phosphate.^18^ (Fig. 2**b**). This increase of GA3P activates the peroxidant pathway which leads to glucose autoxidation and forms hydrogen peroxide. On the other hand, the glucose autoxidation process leads to the formation glyoxal which is an AGE pre-cursor and encourages OS in cells (Fig. 3**a**)..^19^

## 3 Reasons behind endothelial dysfunction carried on by ROS in diabetic vascular problems

Through direct interactions with endothelial cells, angiopoietin-1 (Ang-1), endothelial cell-selective adhesion molecule (ESAM), endothelin-1 (ET-1), and other factors regulate endothelial cell function. Diabetes patients have higher plasma levels of ET-1, a vasoconstrictor peptide produced by endothelial cells. ET-1 overexpression directly prevents NO release and eNOS.^20^ Furthermore, via Nox1, ET-1 overexpression encourages the development of perivascular oxidative stress, inflammation, and atherosclerosis linked to type 1 diabetes.^21^ Vascular endothelial cells produce ESAM, which is a member of the immunoglobulin superfamily. Endothelial permeability, angiogenesis, and tight endothelial junctions are all regulated by ESAM.^22^ Patients with type 2 diabetes had higher serum ESAM levels, which were negatively connected with catalase activity and favorably correlated with malondialdehyde (MDA) levels. Patients with type 2 diabetes who had elevated oxidative stress had greater ESAM levels. As an endothelium-specific protective factor, the activation of TIE-2 by endothelial cells is essential for vascular integrity. Hyperglycemia and advanced glycation products (AGEs), which disrupt Ang-1 and TIE-2 signaling in endothelial cells, induce nuclear translocation and increase Ang-2 in endothelial. This leads to vascular instabilities, inflammatory reactions, and endothelial cell death.^23^, ^24^ It is crucial to protect endothelial damage in diabetic patients. In diabetes, certain oxidative stress inducers can block endothelial factors that are related to endothelial functions by inducing oxidative damage, which leads to endothelial malfunction.

## 4 Type 2 diabetes pancreatic beta (β)-cells failure and insulin resistance

Insulin is a crucial hormone for metabolism regulation. The pancreatic β-cells have an important role in producing and secreting it. Insulin is essential for the regulation of metabolism in key energy sources such as proteins, carbohydrates, and lipids. Insulin helps glucose to be absorbed from the bloodstream into cells. This includes cells in adipose tissues, the skeletal muscles, and liver cells. Understanding the impact of insulin on a wide range of physiological processes, including the production and regulation of the hormone, is important for understanding T2D.^21^ Notably, disturbances in insulin signaling through the inhibition of the insulin receptor substrate protein, phosphoinositide-3-kinase, and protein kinase B (AKT) lead to insulin resistance even when suffering from diseases like cancer.^25^ This mechanism is linked to illnesses like diabetes causing extraordinary harm to several cellular functions.^26^ This process is associated with unprecedented damage to many cellular processes during conditions like diabetes. Excess ROS generation can harm proteins, lipids in cells, and DNA if left unchecked, which would impair metabolic performance.^11^ In preclinical models and human systems, the mitochondrial electron transport chain continues to be the primary source of reactive oxygen species (ROS), despite coming from many cellular compartments. Pancreatic β-cell mitochondria are essential for coordinating the precisely timed release of insulin in response to glucose. They act as the metabolic engines that connect insulin secretion and glucose metabolism. These mitochondria essentially serve as molecular bridges, transforming the energy from the metabolism of glucose into signals that cause the exocytosis of insulin, which controls blood sugar levels.^27^ Extended exposure to high glucose concentrations has been associated with pancreatic β-cell dysfunction, which is marked by a variety of biochemical and molecular abnormalities. These include abnormal activation of protein kinase C, increased production of advanced glycation end products, impaired oxidative phosphorylation, and deregulation of pathways such as the hexosamine and polyol pathways. Collectively, these processes lead to the decline of β-cell performance and ultimately hinder the cell's capacity to efficiently control glucose levels. Any disturbances in mitochondrial function are indicative of impaired metabolic activity, which might cause pancreatic β-cells to die more quickly. This decline in mitochondrial function compromises the cell's metabolic stability, which leads to increased vulnerability to apoptosis and ultimately β-cell loss..^28^

## 5 Different kind of antioxidants sources that related to DM-OS

Natural antioxidants are known as chain-breaking antioxidants, which react with lipids free radicals and convert them to highly stable products. These kinds of molecules have various classes that could directly target the functional implications of DM. In general, these molecules could be as bioactive proteins, polysaccharides, and various kinds of vitamins, flavonoids, carotenoids, and catechins. For instance, vitamins are the vital components that are essential for DM metabolic activities, for example, ascorbic acid (Vitamin C), alpha-tocopherol (Vitamin E), vitamin B, and its subtype.^29^ In addition, hydroxycinnamic derivatives that could be found in a wide variety of food, including wheatgerm, corn, and green leaves, and that could regulate the expression of the necrosis factor-α (TNFα) and increase the secretion of the adiponectin anti-inflammatory from adipocytes which significantly decrease glycohemoglobin (Table 1).^30^ While Vitamin C and Vitamin E antidiabetic functions are influenced by the antioxidants related mineral.^31^ Where, minerals are important cofactors in enzymatic antioxidants. Besides, they are playing an active role during the metabolic process of many macromolecules as important co-factors in the production of antioxidant enzymes. They also play active roles in cellular metabolism, such as carbohydrates, nucleic, and other macromolecules (Table 2). Like selenium (Se) that is conjugated with the phytochemical antioxidant functions like flavonoids that are phenolic antioxidants and catechins from green and black teas and sesame oil.^32^ Where Se is essential for glutathione peroxidase (GPx) enzyme activity that directly associate with diabetes stress due to its significant impact on the cellular ROS.^32^, ^33^ In a clinical study showed a significant increase in GPx antidiabetic activity according to Se levels in the blood according to 50 diabetic patients and 50 healthy adults..^33^

**Table 1:** Natural Antioxidants with chemical formula.

Natural Antioxidants	Natural sources (*Scientific name*)	Chemical Formula	Antidiabetic mode of action	Ref.
Hydroxycinnamic Acid	Wheat cereal (*Triticum aestivum* L)		Reduces the expression of the necrosis factor-α (TNFα), monocyte chemoattractant protein-1 (MCP-1), and plasminogen activator inhibitor type-1 (PAI-1), and increases the secretion of the adiponectin anti-inflammatory from adipocytes.	^30^
Vitamin E	Canola (*Brassica napus*)		Reduces the insulin resistance, reduced glutathione (GSH); and quantitativly increases the insulin sensitivity Index.	^31^
Quercetin	apple, (*Malus domestica*)		Inhibits GLUT2 that reduces the absorption of glucose in small intestine and block the activity of tyrosine kinase Improve GLUT4. Reduces the lipid peroxidation genes.	^34^
Resveratrol	Peanut (*Arachis hypogaea*)		Phosphorylates protein kinase B (pAkt): protein kinase B (Akt) ratio in blood platelets and enhance the β‐cell function through its adverse effects.	^35^
Catechin	Green tea (*Camellia sinensis*)		Regulates transporters related to the absorption of glucose, including SGLT1, GLUT2, and GLUT5.	^36^
Hydroxybenzoic Acid / Gallic acid	Pomegranates (*Punica granatum*)		Attenuates insulin resistance via regulating the miR-1271/IRS/PI3K/AKT/FOXO1 pathway and thus affecting protein expressions involved in insulin signaling.	^37^
β-carotene	Carrot (*Daucus carota* L)		Inhibits cell transcription factors, such as NF-κβ and inflammatory cytokines that are involved in the distribution of adiposity and insulin resistance.	^38^

**Table 2:** Antioxidant mineral and their mechanism of actions.^32^, ^33^, ^39^

Minerals	Natural sources (Scientific name)	Symbol	Antidiabetic mode of action
Boron	Grape (*Genus Vitis*)	Br	Boron treatment repressed the expression of adipogenesis-related genes and proteins by regulating β-catenin and AKT showing an antioxidant effect on the pancreatic β-cells.
Cobalt	Garlic (*Allium sativum* L.)	Co	Cobalt chloride (CoCl_2_) decreases the gluconeogenesis pathway.
Chromium	Broccoli (*Brassica oleracea*)	Cr	Improves insulin binding, receptor number and insulin receptor enzymes by increasing insulin sensitivity, β-cell sensitivity and insulin internalization.
Selenium	Yeast (*Saccharomyces cerevisiae*)	Se	Glutathione peroxidase (GPx) enzyme activity that directly associate with diabetes stress due to its significant impact on the cellular ROS. In a clinical study showed a significant increase in GPx antidiabetic activity according.
Zinc	Sesame (*Sesamum indicum* L.)	Zn	It affects the production of mitochondrial antioxidants due to the inhibition of α-ketoglutarate dependent mitochondrial respiration.

In addition, flavonoids that are linked with the mineral’s levels and that have different classes like flavones, flavonols, flavan-3ols, isoflavones, anthocyanidins, catechins and flavanones are important phytochemicals that could regulate DM-OS.^34^, ^35^, ^36^ Where, their reduce glycemia action mechanisms could be from inhibiting the aldo–keto reductase (AKR)1B, an NADP(H)-oxidoreductase like incase of linarin, isorhamnetin, and isorhamnetin.^34^, ^35^ In addition, quercetin, resveratrol, and catechin flavonoids present numerous DM related functions from inducing the function of the PPARγ signaling and suppressing CD38; that increase insulin sensitivity, reduce glucose tolerance and glycohemoglobin A1C formation in consequences.^36^, ^37^.

Regarding these antioxidant chemicals mechanism of action and administration dosages, in 2016, the U.S. Food and Drug Administration (FDA) released the most recent nutrition facts label (NFL) on packaged food based upon scientific evidence, including the relationship between diet and chronic illnesses, like obesity and heart diseases. Like incase of carotenoids that are fat-soluble pigments found in carrot and mango and lycopene phytoconstituents in tomatoes.^39^ These pigments have been linked to antidiabetic properties. Where, the FDA has reported these molecules represented in β-carotene with Vitamin C, and Vitamin E as antidiabetic agents.^31^, ^38^, ^39^ Besides, the evidence of the antioxidants in preclinical studies showed that the high dosage of Vitamin C with doxorubicin has induced the resistance to treatment of the chronic myelogenous leukemia (K562) and lymphoma (RL) in mice.

## 6 Relationship between antioxidants and the development of AGE (Carbonyl stress product) as a main factor related to DM

The glucose levels at hyperglycaemic states is autoxidized to form the carbonyl-containing molecule, glyoxal, which is an AGE pre-cursor, is formed. Non-enzymatic dephosphorylation of glucose metabolites such as GA3P and dihydroxyacetone-3-phosphate produce methylglyoxal, another precursor of AGE. The AGE receptors AGE-R1, AGER3, AGER2, and AGER4 are activated by glyoxal or methylglyoxal.^1^ The third AGE precursor is 3-deoxyglucosone, which is generated by the glucose-produced adduct of lysine 1-amino-1-deoxyfructose which is also known as the Amadori product.^40^ Additional matrix elements that are also nucleic acid and lipids, have been demonstrated to be capable of changing into AGE.^41^, ^42^ (Fig. 3**b**). In general there are three ways for the intracellular synthesis of precursors of AGE that can harm the cells. In the first place, proteins in intracellular synthesis that were altered by AGE function in different ways. Secondly, by AGE precursors, extracellular matrix elements are altered in aberrant relations with other matrix elements and cell surface-expressed matrix receptors (integrins). Finally, AGE receptors on cells including macrophages, vascular smooth muscle cells, and vascular endothelial cells connect to plasma proteins changed by AGE precursors.^41^ In the event of activation, RAGE (receptor for advanced glycation endproducts) receptors trigger a series of cell-mediated events that trigger the production of ROS. In turn, these ROS molecules are cellular messengers, activating a series of actions that lead to the activation of a multi-faceted transcription factor dubbed nuclear factor kappa B (NFkB). This activation, in turn, sets in motion a complex series of alterations in the regulation of genes, leading to a multitude of pathological modifications in cellular gene expression. Also, the elevated levels of methylglyoxal produced by endothelial cells and metabolites in the kidneys of mice promote alteration to mSin3A.^39^, ^40^ Sp3 becomes more susceptible to N-linked O-Glc glucosamine modification after the methylglyoxal methylation of mSin3A. Increased Ang-2 expression is the result of Sp3′s altered ability to bind to a glucose-responsive GC-box in the angiopoietin-2 (Ang-2) promoter.^41^ The improved expression of Ang-2 has led to the development of endothelial microvascular cells more prone to anti-inflammatory actions of tumor necrosis factors alpha (TNF) in kidneys and cells of patients with diabetes mice. In addition, it's been discovered that methylglyoxal alters the 20S proteasome which reduces its activity in the kidneys of diabetics *et al* and decreases the polyubiquitin-receptor 19S-S5a. These findings suggest the possibility of a new relationship between hyperglycemia and cell dysfunction..^42^

In that context, several antioxidants in nature nonenzymatically inhibit the Glycation process by stabilizing the protein structure.^43^ In the end, the helix structure is stabilized by the spontaneous hydrogen bonding with proteins and van der Weals forces. Natural Antioxidant Types and Their Roles in Diabetes Mellitus (DM) Complication Prevention. Like catechin that presents in tea herb and that has a high antidiabetic property according to its strong antioxidants mechanisms from neutralize free radicals, prevent lipid oxidation, and induces the antioxidant enzyme activities. Another example is the carotene that found in carrots, tomatoes, and leafy greens. Where, its protection mechanism for the cell membrane from neutralization of the singlet oxygen of the ROS radicals. Another important example is scavenging of free radicals by Vitamin C from orange fruit (*Citrus sinensis*) that is known to reduce the amount of protein oxidation.^44^ Its mode of action occurs by eliminating oxidative free radicals, preventing advanced glycation end-products (AGEs) formation, and improving the endothelial function. Additionally, astaxanthin inhibits protein glycation by scavenging reactive oxygen species. A recent study showed that sulforaphane can suppress RAGE expression due to its antioxidant properties..^45^ This may represent a novel therapeutic approach for diabetics and for reducing AGE levels. Along with, dicarbonyl compounds, especially active ones and dicarbonyl compounds containing a carbonyl group are important precursors for AGEs. The carbonyl functional group on the adjacent carbon atoms results in a high degree of reactivity. The trapping of dicarbonyl compounds by many natural compounds is significant and can inhibit the formation of AGEs in a highly dose-dependent manner. In the majority of studies, polyphenols prevent AGE formation through a variety of mechanisms, including chelating metal ions to capture active carbonyls, covering glycation sites of proteins, and lowering glucose levels in blood..^46^ Fig. 3**c** illustrates the formation of reactive carbonyl species and shows reactions of protein glycoxidation.

## 7 The role of antioxidants on enhancing DM hexosamine pathway flux

Under glycemia, fructose-6-phosphate (F-6-P) levels rise, and by glucosamine-fructose aminotransferase, the molecule is broken down to glucosamine-6-phosphate, after which it is converted to uridine phosphate-N-acetylglucosamine (UDP-GlcNAc) via the movement of UDPN-acetylglucosamine-1-phosphate uridyltransferase. O-glucosamine-N-acetyltransferase is activated by UDP-GlcNAc accumulation, and this process is connected to the prooxidant function of the hexosamine pathway. The action of this enzyme and the hexosamine pathway is connected to alterations in the expression of genes and amplified expression of TGF-a and TGF-b (transcription factors), which suppress mesangial cell mitogenesis, promote collagen matrix proliferation, and thicken the basement membrane..^47^

The transcription of important genes like TGF-a, TGF-b1, and PAI-1 is elevated by the onset of the hexosamine pathway through hyperglycemia. It's been established that the condition of hyperglycemia results in a fourfold increase in the Sp1 transcription factor's O-GlcNAcylation; in TGF-b1 cells of the vascular smooth-muscle and PAI-1 within endothelial cells of the arterial wall, it is responsible for the induction of hyperglycemia, which then triggers the PAI-1 promoter..^48^

**Fig. 4:**
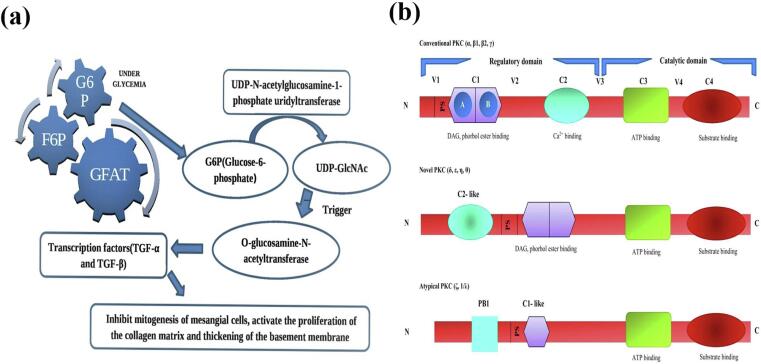
**(a)** Pathways of increased hexosamine pathway flux**. (b)** Schematic presentation of the domain structure of PKC isoforms.

O-GlcNAcylation inhibits the activity of endothelial nitric oxygen synthase at the Akt-activated site on eNOS. This is relevant to diabetic vascular complications in arterial endothelial cells..^49^ In addition, hyperglycemia increases the activity of GFAT in aortic smooth muscle cells, and it increases the O-GlcNAc-modification of numerous proteins.^50^, ^52^ (Fig. 4**a**).

Due to the significance of OS in a variety of DM issues, numerous studies have been conducted to determine the antioxidant properties of a wide range of substances, which include naturally occurring antioxidants from plants..^51^ Specific data on the importance of antioxidants (like glutathione and coenzyme Q10 and a-lipoic acids) to reestablish insulin sensitivity have been gathered..^52^ The study revealed that- and g-tocopherols in combination with retinol, cryptoxanthin, and g.^53^ ascorbic acid^54^ and carotene and b-carotene the zeaxanthin compound and lutein^55^ and lycopene^56^ significantly reduces DM complications. The study revealed that the phytochemical constituents (>10,000 substances found) from a variety of plant species, both medicinal and food-based, possess powerful anti-radical and anti-inflammatory properties. They alter the actions of enzymes, such as −glucosidase or lipase. They reduce the amount of glycemia and increase pancreatic functions, providing the synergistic effects of medications for hypoglycemics which makes them extremely efficient in treating diabetes..^57^

## 8 PKC activation pathway and antioxidant potentials

At least eleven PKC isoforms which belong to the family known as “PKC” that is found in mammals' tissues. The standard isoforms' activity is dependent on phosphatidylserine Ca^2^^+^ ions, and diacylglycerol (DAG) which significantly increases this action.^58^ The chronically high DAG concentrations are caused by the increase of dihydroxyacetone phosphate, a glycolytic intermediate in hyperglycemic conditions or diabetes. This intermediary is converted to glycerol-3-phosphate, which then promotes the de novo synthesis of DAG. Total DAG levels in vascular tissues are increased in diabetes such as the retina^59^ aorta, heart,^60^ and renal tissue.^61^ And in various nonvascular tissues such as the liver and skeletal muscle.^62^ Yet, it's important to note that there is no uniform alteration observed in the levels of diacylglycerol (DAG) within both the central nervous system and peripheral nerves.^63^ Many studies have shown that DAG increases in a cell culture in a time-dependent manner. The amount of glucose in the cells rises from 5.5 min to 22 min,^59^ renal mesangial cells^60^ and smooth muscle cells.^61^ Belonging to the AGC enzyme family (including cAMP-dependent protein kinase and protein kinase G and protein kinase), PKC is an influential serine/threonine-related protein kinase. This protein plays a crucial role during the cell process, and it has an impact on many pathways that are associated with the transmission of signals.^62^ PKC contains several forms that serve different biological systems^63^ (Fig. 4**b**).

Phosphatidylserine (PS), calcium, and diacylglycerol (DAG) or specific compounds like phorbol 12-myristate 13-acetate (PMA) are the triggers that activate the conventional isoforms of PKC (cPKC) PKC-α, −β1, −β2, and −γ. Distinctly, the original isoforms of PKC (nPKC), including PKC-δ, −ε, −θ, and −η, are brought into action by phosphatidylserine (PS), diacylglycerol (DAG), or agents like phorbol 12-myristate 13-acetate (PMA). However, it's noteworthy that calcium does not partake in the activation process of these novel PKC isoforms.

Distinctly, the novel isoforms of PKC (nPKC), including PKC-δ, −ε, −θ, and −η, are brought into action by phosphatidylserine (PS), diacylglycerol (DAG), or agents like phorbol 12-myristate 13-acetate (PMA). However, it's noteworthy that calcium does not partake in the activation process of these novel PKC isoforms. The role of the antioxidants in this process come from scavenging radicals and inhibiting signaling enzymes like protein kinase C (PKC) that could play an important role in diabetes complications. PKC has different regions that are susceptible to oxidation, which allows it to respond to antioxidants in a way to causes opposite cell responses. PKC is activated by oxidant diabetic promoters that react with zinc-thiolates within the regulatory domain. The oxidized form of antioxidants causes oxidation, which is the same target cell that diabetic promoters bind to, and inactivates PKC. It may be possible to create a counter-active mechanism that blocks signal transduction. This may be at least partly responsible for the antioxidant-induced prevention of diabetes complications, and for inducing cell death.^64^.

It is worth noting that phytochemicals like anthocyanins and polyphenols are antioxidants because they work because they stop their production of prostaglandins and proinflammatory cells, transcription factors in particular NF-kB factor.^65^ Curcumin can aid in the management and decrease of the likelihood of DM problems thanks to its anti-inflammatory and antioxidant effects, Butein is an antioxidant polyphenol that inhibits NO production in the laboratory. It guards the B-cells of the pancreas from excessive inflammation. It could be used to lessen the intensity of DM1.^66^ Resveratrol regulates the expression of genes closely associated with DM2 advancement by influencing the level of expression for several cells' genes and insulin production in pancreatic cells.^67^ In the laboratory under laboratory conditions, the effect of antioxidants in the DM process was examined. Thus, the inclusion of coenzyme Q10 with L-arginine aided in reducing the effects of OS and increasing the NO concentration and effectiveness against the NO synthesis inhibitor, NO-nitro-L-arginine methyl ether. It also eliminated any effect the inhibitor had on the LPO-AOD indicators and the NO concentration..^68^

## 9 Various biological targets of PKC (Protein kinase C) isoform activation

**Fig. 5:**
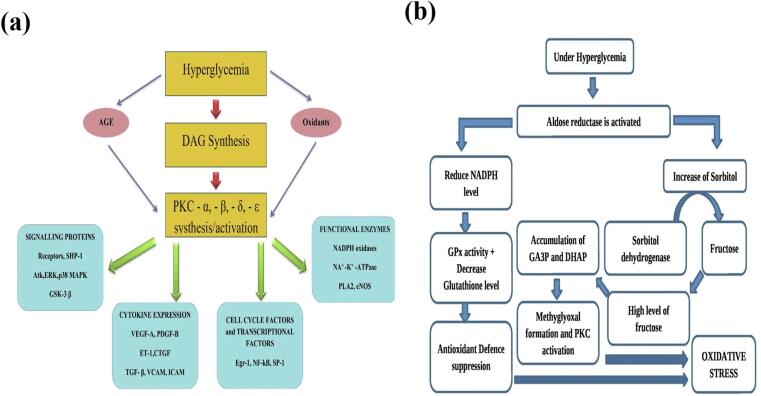
**(a)** Various biological targets of PKC isoform. **(b)** Schematic diagram of increased polyol pathway flux.

PKC fundamental activation has been the focus of extensive and exemplary evaluations that were published. About the present state of knowledge in this area the focus of our research will be on investigating the effects of elevated levels of glucose on the triggering of PKC. Additionally, PKCs have the potential to be spurred into action by oxidizing agents like hydrogen peroxide (H_2_O_2_), operating in a manner distinct from their response to lipid-based secondary messengers^69^ and increase in mitochondrial superoxide due to high levels of glucose.^70^ Diabetes-associated alterations in various PKC isoforms give rise to a plethora of irregular vascular and cellular phenomena. This includes disruptions to endothelial function and changes in vascular permeability, irregular angiogenesis, asymmetric cell growth, and programmable cell death changes in the dilation of vessels, the basement membrane's thickness, and growth of the extracellular matrix. The enzyme activities of key components like mitogen-activated protein Kinase (MAPK) and cytosolic Phospholipase A2 (PLA2) and Na + K+ATPase and shifts in the transcription of multiple factors are the main factors behind these changes (Fig. 5**a**).

The increased levels of glucose cause activate PKC isoforms by three distinct routes: firstly, through the stimulation of diacylglycerol (DAG) synthesizing; and secondly, it promotes the production of advanced glycation products (AGEs) and thirdly through the increase of the level of oxidative stress inside cells. When PKC activates in a cell, it initiates an array of actions which can cause a variety of problems. These include retinal neuronal, renal, and cardiac concerns and PKC controlling the release of the signaling proteins, cytokines, and functional enzymes which collectively can contribute to developing these issues.

After evaluating the positive impact of the synthetic biguanide precursor N-[imino(1-pipe pyridinyl)methyl]guanidine on free radical homeostasis, coenzyme Q10′s beneficial effectiveness became apparent.^71^ Green tea ingredient epigallocatechin gallate is particularly effective in OS parameters and plasma antioxidant capacity. Our study showed that the utilization of N-acetylcysteine can be appropriate in patients suffering from symptoms of DM2 that were aggravated by microangiopathy in the lower extremities.^72^ This drug could result in a decrease in the indicator of carbonyl stress as the result of a drop in methylglyoxal, Glyoxal *et al*so the ability of the cell to maintain its ability to redox and the increase in cysteine levels and levels of glutathione and a decrease in the proportion of the oxidized. Among domestic medications, ethylmethylhydroxypyridine malate and ethylmethylhydroxypyridine succinate should be highlighted since they have antioxidant, antihypoxic, and membrane-protective characteristics against vascular DM complications.^73^ In the same way, there are conflicting findings about the absence of significant therapeutic benefits of the most known anti-oxidant therapies, especially for patients suffering from DM2. This may be due to the complexity of the disease and the fact that it is only certain treatments share similar properties and goals, or the immutability of some changes with a variety of consequences..^74^

## 10 Regulating the polyol pathway flux through antioxidant administration

The family of enzymes known as aldo-ketoreductase is at the heart of the polyol pathways. The enzymes can utilize many carbonyl-based compounds as substrates and decrease them through the nicotinic acid dinucleotide phosphate (NADPH) to the corresponding sugar alcohols (polyols). It was originally thought that the aldose reductase glucose enzyme converts to sorbitol using sorbitol and transformed into fructose via the enzyme sorbitol-dehydrogenase (SDH) when it is in the presence of NAD+as a cofactor. hyperglycemia. In this condition, aldose reductase is stimulated, which results in an increment in the volume of sorbitol. It transforms into fructose through sorbitol dehydrogenase. High fructose levels cause GA3P and DHAP build-up, which leads to OS via methylglyoxal production and PKC activation.^11^ Many of these organs have insulin-independent GLUTs that mediate glucose absorption; thus, concentrations of intracellular glucose rise in tandem with hyperglycemic conditions. There are a variety of theories in the context of how high glucose levels cause damage to tissues in the pathways of polyol. Most often, it is the rise in redox stress as a consequence of NADPH consumption. NADPH plays a crucial role in the regeneration of glutathione, and GSH acts as a powerful agent to remove ROS. This can cause an increase or decrease in intracellular oxygenation. During the expression of genes that control glutathione regeneration, excess production of aldose reductase in humans improved atherosclerosis in diabetic mice.^75^ GPx activity, and glutathione level, are decreased by aldose reduction. AOD (Antioxidant defense) is suppressed resulting in OS.

The natural antioxidants can decrease oxidative stress-induced diabetic complications by direct scavenging of ROS & by inhibiting cell proliferation secondary to the protein phosphorylation. Antioxidants decrease the accumulation of GA3P and DHAP and block the formation of OS On the other hand by increasing glutathione levels and decreasing GPx activity effects of antioxidants will be activated which will decrease the formation of OS in diabetic complications (Fig. 5**b**). The current therapeutic options for treating diabetes must incorporate antioxidants, innovative delivery techniques like nanoparticles, microparticles, or liposomes, and the making of drugs that affect the sources of ROS and the expression of genes. In the case of drug administration, it is via the microparticles that can provide antioxidants having lower permeability to the membrane (such as SOD). In the research, the encapsulation of SOD caused a growth of 60 % in the production of superoxide, whereas SOD that was free SOD produced only a slight influence. This suggests that modern therapies for the treatment of diabetes must comprise antioxidants, novel ways of distribution, including nanoparticles, microparticles, or liposomes, and the creation of drugs that alter the source of ROS and the modification of gene expression. The administration of medications as a microparticle-based device could help in the provision of antioxidants with minimal membrane permeability (such as SOD). It was found that incapsulation of SOD caused a reduction of superoxide production by 60 in comparison to the unencapsulated SOD resulting in only a slight effect. Another study found that curcumin has more potent antioxidants when placed within liposomes (artificial bilayers of lipids).^76^ These new antioxidant delivery systems offer great possibilities for use as therapeutics to treat the disease of diabetes. Indirect exposure to ROS sources −the creation of specific antioxidants is a different type of revolutionary treatment method that has led to important progress in the management of DM and its ramifications. MitoQTPP and TEMPOL is a mitochondrial antioxidant that is used to reduce OS and increase the likelihood of a good prognosis for diabetic patients..^77^

## 11 Antioxidants and the deactivation of insulin signaling pathway

**Fig. 6:**
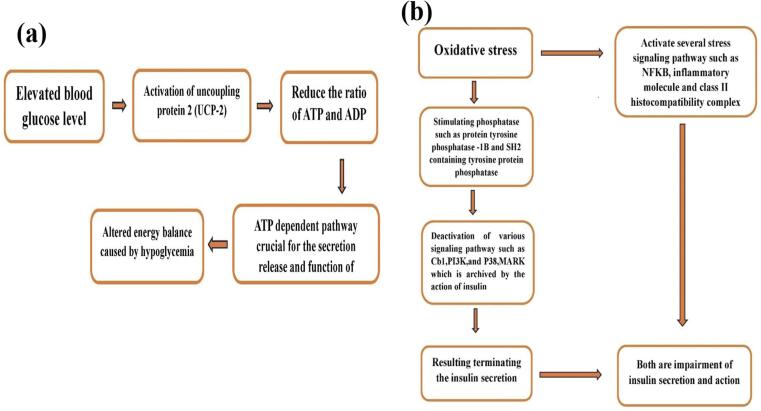
**(a)** High glucose induces energy balance alteration. **(b)** Schematic diagram of oxidative stress impairment insulin secretion and action.

The elevation in blood sugar levels triggers the activation of the protein uncoupling-2 (UCP-2). This is subsequently, a cause of a decrease in the proportion of Adenosine Triphosphate (ATP) to diphosphate adenosine (ADP). In turn, the efficacy of ATP-dependent pathways essential to the release, secretion, and functioning of insulin gets affected due to the altered energy balance that is caused by hyperglycemia.^78^ (Fig. 6**a**).

OS regulates the activation of the signaling pathways that are usually activated in response to insulin-related actions, including Cb1, PI3K, and MAPK p38. It is thought that the mechanism of deactivation includes activating enzymes like the SH2-containing protein-tyrosine phosphatase and the protein tyrosine 1-B enzyme. The result of these events is the reduction of insulin's effect and effect.^79^ OS also triggers the induction of multiple signals that respond to stress. These pathways include components such as the NF-kB. Within these pathways, the presence of inflammation-related agents, like the inducible synthase of nitric oxide, and components such as histocompatibility complexes of class II are heightened because of the stress-induced oxidative activation.^80^ In general, these pathways can hurt insulin's action and release (Fig. 6**b**).

ROS-induced damage to oxidative cells in cells of the β-cells that are triggered by high glucose levels affects the volume and the quality of released insulin.^81^ Existing evidence supports the importance of dysfunction caused by oxidative stress within Β-cells. The dysfunction is manifested as a decreased production capacity, accompanied by increased insulin resistance. This interaction between the oxidative stress of Β-cells and their dysfunction plays a crucial role in the pathogenesis of the two types of diabetes Type 1 and Type 2. Mellitus.^82^ The shape, size, and functions of mitochondria may change as a result of excessive ROS-generating cells that can cause the degeneration of K+channels dependent on ATP and a decrease in insulin production. The mechanisms behind this could be related to the distinct difference in the amount of antioxidant enzymes in Β-cells. As compared to the cells that are found in organs, such as kidneys, livers and the heart and brain and the heart, the number of antioxidants in Β-cells is significantly less and ranges from 10-to-20 times less. The amount of GPx and catalase within the liver ranges from 5 % to 5 percent. It was found that mitochondrial Mn-dependent SOD2 and as well with the Cu/Zn-dependent cytoplasmic SOD1 genes are not sufficient to provide 50 % of the volume of production in the liver.^83^ Endothelial dysfunction is among the most prevalent issues of DM and is caused due to the activation of a variety of other pathways via ROS. Endothelial dysfunction may be a factor in the proliferation of leukocytes, the adhesion of platelets to thrombosis, and inflammation reactions. They are among the major components of atherosclerosis. It is a significant risk factor for the cardiovascular effects associated with diabetes.^84^ (Fig. 6**b**).

It was found that short exposure to high glucose levels triggers the production of NO to increase and an upregulation selectively of the inNOS gene. When superoxide radicals are increased, the production of peroxynitrite is also elevated. This powerful oxidant can have a detrimental effect on vascular links, which may exacerbate the disease and cause myocardial injury.^85^ The increase in the number of NOX isoforms found in monocytes and macrophages. In addition, the production of ROS and stimulation of the production of pro-inflammatory proteins such as monocyte chemoattractant proteins 1 (MCP-1) and Intercellular adhesion molecules 1 (ICAM-1) are all triggered by activation of the PKC and AP-2 and AGE generation. Monocytes, macrophages, and vascular cells all express different isoforms of NOX, which both play a defensive role and help to form endothelial dysfunction and inflammation..^86^

The development of neuropathy, nephropathy, and retinopathy, three more severe DM consequences, is significantly influenced by OS. NOX enzymes specifically, NOX4 and NOX5 homologs are the primary ROS production in kidneys. Many variables, such as the NF-B p65 NF-B subunit, TNF, TGF, and fibronectin affect the function and expression of the enzymes leading to proinflammatory as well as profibrotic signs to increase.^87^ The most prominent molecular reason for diabetes-related retinopathy is glucose which also influences the polyol-hexosamine pathways PKC, RAGE/AGE axis metabolism pathways. OS lowers the amount of hypoxia-induced factors alpha (HIF1) within endothelial retinal cells that promote angiogenesis.^88^ In the case of diabetic neuropathy OS results from an elevated glucose level that causes nerve cell death via lipid peroxidation (LPO) and DNA damage which can trigger pathological repair pathways, a decrease in antioxidants in cells, well as activation by proinflammatory transcription factors..^89^

Genes that are redox-sensitive and linked to antioxidant defense (AOD), and genes whose activation is influenced by high concentrations, are significantly stimulated when antioxidant processes are elevated. The promoters of these genes have special binding sites specifically designed to accommodate important transcription factors, such as Nf-kB, AP-1 and Nrf2, FoxO, PPARS, and Bach 1. The coordinated interaction of the transcription factors mentioned above, specifically in the NF-kB/ARE systems, is intricately controlled by the interaction between the development of inflammation and antioxidant enzyme activity.^90^ In the process of generating Nrf2 and NFB transcription factors, the proteins, which are referred to as insulin-dependent effector proteins (Akt Kinase, MARK) and insulin, regulate the activities of antioxidant enzymes. Despite the fervent research into the significance of oxidative stress reaction as a factor that causes the process of developing DM and the complications that accompany it, currently, the most important thing is spotting crucial biochemical markers for free radicals, together and resolving the relation between oxidative stress-related reactions and inflammation and carbonyl stress as part that of “metabolic memory” phenomenon that could be employed as a secondary indicator for monitoring the progression of disease.

## 12 The role of antioxidants on the insulin resistance and atherosclerosis

The majority of type 2 diabetics suffer from the effects of insulin resistance (IR) can affect the vast majority of people and the two-thirds who have impaired tolerance to glucose. Both of these categories are much more likely to develop cardiovascular disease (CVD).^91^ Superoxide overproduction produced through free fatty acids (FFAs) triggers several proinflammatory signals and deactivates two crucial antiatherogenic enzymes, prostacyclin synthase, and eNOS, through similar processes as ROS induced by hyperglycemia. Through inhibition of FFA release from the adipose tissue, inactivation of prostacyclin synthase and eNOS was prohibited, by the inhibition of the rate-limiting enzyme, carnitine palmitoyltransferase I, which develops when superoxide levels are lowered and is in charge of the oxidation of fatty acids in mitochondria..^92^

**Fig. 7:**
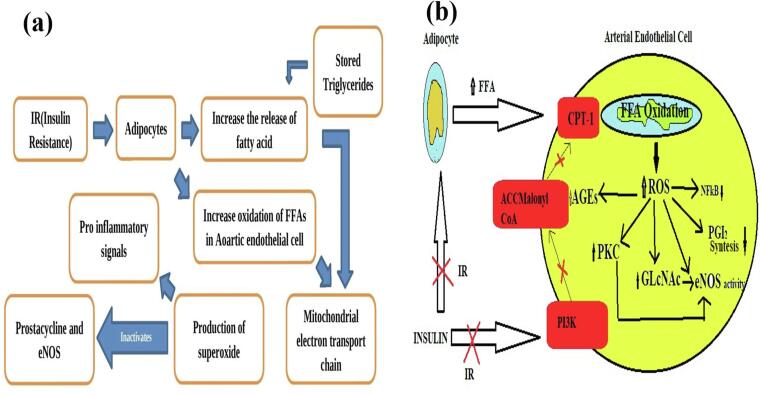
**(a)** Insulin resistance, ROS, and atherosclerosis. **(b)** The role of insulin resistance and free acid fatty acids in the macrovascular ROS-forming cell and thermogenesis.

Collective insulin levels to get rid of hyperglycemia induced by pathway-selective insulin resistance which is likely to activate insulin signaling pathways that are non-resistance in nature, including MAPK (Mitogen-activated protein kinase). Such specific activation of the MAPK pathway by insulin would encourage cellular development and relocation as well as the production of prothrombotic and profibrotic substances in artery endothelial cells. The hormone insulin stimulates the production of vasoconstrictor endothelin-1 (ET-1) which increases the number of adhesion molecules. A high level of insulin can stimulate VSMC growth and migration in addition to the creation of angiotensinogen and AT1R within blood smooth muscle cells by taking over the MAPK pathway.^93^ (Fig. 7**a**).

It is worth noting that the natural antioxidants could block various transcription factors such as NFkB, AP-1, and HIF-1. It will also decrease gene expressions (IL-6, TNF-α), and will prevent the formation of diabetes complications, by preventing insulin resistance. By blocking the activation of PI3K, increases Glut 4 transport, and causes glucose metabolism which also prevents insulin resistance. By increasing the activity of carnitine Palmitoyltransferase, the enzyme that limits the release of FFA from adipose tissues, the inactivation and inhibition of eNOS, and prostacyclin synapse, was prevented and the mitochondrial electron transportation process is also blocked by these natural antioxidants (Fig. 7**b**).

## 13 Experimental studies of the antioxidant enzymes implications on the DM oxidative stress

Alloxan (AX) and streptozotocin (STZ) diabetes non-genetic models are considered the most popular, readily available as well and easily repeatable techniques for studying OS response. AX as well as STZ are structural sugar analogs, specifically aggregated in pancreatic β-cells, and can bind with the sugar transporter GLUT2 and cause ROS damage to cells. These diabetogenesis are used to simulate DM1 at a variety of concentrations.^94^ The various experiments in modeling AX DM have shown to be relevant in the state of the LPO AOD as it changes in biochemical variables which account for detoxification function in rodents. In the end, chemiluminescence research revealed that the LPO-AOD system is involved in the processes of blood from animals, and the heart, kidneys, and liver became more intense. Thiol group counts declined and confirmed that the restoration of antioxidants with low molecular weight is affected when under AX trigger DM conditions.^95^ It was found that the abnormalities in the metabolism of lipids and proteins and excessive glucose and LPO activation are the most atherogenic.^96^ Changes in both the antioxidant and pro-balance and the decrease in catalase and SOD activity in serum from blood were observed on the 10th day of the AX DM process. This is characterized by the presence of hyperglycemia. The cytotoxicity associated with AX has been proven to be triggered by the activities of free radicals and the oxidation process of SH-groups in proteins that result in necrosis, as being caused by problems with calcium homeostasis, and destabilization of mitochondria's membranes, which is monitored via caspase cascade stimulation, without involvement of the p53 protein (which performs apoptosis).^97^ For DM modeling, the STZ DM model is commonly utilized. There is evidence that the STZ DM model has more validity since OS responses are more intense and animals live longer. For example, in the STZ DM model, there was a marked initiation of LPO reactions, modifications to energy metabolism (inhibition of aerobic ATP synthesis, accumulation of lactate, dissociation of oxidative phosphorylation, and the onset of lactic acidosis), and modifications to the useful state of cell membranes (structural rearrangement of lipid's membrane), microviscosity disorders, and a reduction in movement of insulin receptors and membrane-bound Na+, K-ATPase and Ca^2+^-ATPase. Recent research has shown that even in the beginning phases of STZ-induced DM in rodents, LPO intensity rises together with hypercholesterolemia and hypertriglyceridemia. This is in the context of reduced antioxidant enzymes and reduced levels of o-3 acidic acids. It was discovered that when the conditions are in a laboratory, OS increases and the amounts of total NO components in the blood of animal serum drops.^98^ Ventricular cardiomyocytes, especially left ventricular cardiomyocytes of rats with DM both alone and in conjunction with arterial hypertension had significantly lower levels of the molecular chaperone HSP60, which is vital for protecting against OS. An improved look at the genes for SOD2 and catalase has a significant inhibitory effect on streptozotocin-induced hyperglycemia, suggesting that important ROS are involved in cell dysfunction.^99^ The reaction of antioxidant enzymes (SOD catalase, SOD, and glutathione enzymes) which are vitally required in the development of OS in the laboratory of DM1 is incredibly indefinite and is not based on any species of animal. This should be emphasized. The time in DM1 is the most important element that affects the way these enzymes perform; at the very first stages of DM1 the body experiences a counter-acting increase in the activity of enzymes, and this will be followed by depletion at the end. In addition to catalase and GPx deficit, increased SOD activity across a variety of tissues, such as the skeletal muscles, the heart, and kidneys and the liver are evident at the beginning phase of DM1. There is evidence that xanthine dehydrogenase mRNA altered xanthine activity are the main cause of OS caused by the hyperglycemia that occurs in various types of fat tissue. Different types of DM2 models and the polygenic nature of the condition are most likely to cause the diversity of the LPO-AOD procedures. Particularly, an exclusive model that takes into consideration the animals' species, sex, and tissue type is used to determine the level of antioxidants in the experimental DM2. However, there was a consistent pattern that developed in which as the severity of diabetes issues grew, OS increased and AOD diminished. At the beginning of DM, it is known that the use of antioxidants and antidiabetic drugs changed the state of animals, and restored the activities of the enzyme portion that is involved in the AOD process.^100^ Recent research has revealed that the underlying characteristics of the various phenotypes that are associated with the human condition of diabetes cannot be replicated in animal models of diabetes cardiomyopathy, atherosclerosis, and other types of diabetic macrovascular disorder.^101^ Large animals such as humans or pigs have been used as models for various studies due to their cardiovascular problems due to diabetes. Further testing of ROS within these models is the subsequent step.

## 14 Hepatic damage induction by DM and controlling its oxidative stress by antioxidants

Reactive oxygen species (ROS) generation and the body's antioxidant defenses are not balanced, which leads to oxidative stress. These ROS, which include hydroxyl radicals, hydrogen peroxide, and superoxide anions, are the results of several vital biological activities. For example, ROS is essential for immunological processes such as the removal of microorganisms by macrophages and phagocytes, as well as for redox signaling pathways inside cells. Even while ROS are essential for cellular function, an excess of them can overpower the body's antioxidant defenses, resulting in oxidative stress and possible cellular component damage.^102^ Apart from its advantageous functions, oxidative stress plays a critical role in the emergence of several chronic illnesses, including cancer, diabetes, neurodegenerative disorders, and cardiovascular problems. When oxidative stress is produced excessively, it sets off a series of negative consequences that permanently alter vital biomolecules such as proteins, DNA, and lipids. This alteration compromises the integrity and functioning of the cells, which plays a major role in the initiation and development of several pathological processes linked to chronic illnesses.^103^ In terms of diabetes, elevated ROS and hyperglycemia harm the β-cells in the pancreas, which leads to type 1 DM. In addition, the primarily originated ROS were from activated Kupffer cells which are specialized hepatic macrophages found in the liver. These cells generate ROS predominantly through the activities of NADPH-oxidase or inducible nitric oxide (NO)-synthase enzymes. This ROS production by Kupffer cells represents a significant source of oxidative stress within the liver environment, contributing to various physiological and pathological processes in hepatic biology..^104^

In the context of hepatic health, dysfunction in Kupffer cells stands at the forefront of hepatic injuries and contributes significantly to the development of non-alcoholic fatty liver disease (NAFLD) in diabetes mellitus (DM) patients. Despite this vulnerability, the liver boasts an intricate and potent array of antioxidant defenses, which include enzymes like superoxide dismutase (SOD), catalase (CAT), and a suite of glutathione (GSH)-related enzymes such as glutathione-S-transferase (GST) and glutathione peroxidase (GPX). These defensive mechanisms work diligently to scavenge free radicals and neutralize hydrogen peroxides, thereby shielding liver cells from oxidative harm. For instance, various studies have underscored the critical role of SOD and CAT activities, highlighting their reduction in hyperglycemic conditions, which consequently escalates oxidative stress and contributes to liver injury. Moreover, glutathione (GSH) emerges as a pivotal endogenous antioxidant, thanks to its thiol group, which undergoes oxidation to produce glutathione disulfide, thus bolstering cell viability. Notably, GSH plays a central role in maintaining the equilibrium of the GSH-related enzyme family. Diminished GSH levels observed in diabetic rat livers correlate with reduced activities of GST, GPX, and glutathione reductase, ultimately leading to the accumulation of oxidative stress. This intricate antioxidant network underscores the liver's remarkable resilience against oxidative insults, highlighting its pivotal role in maintaining hepatic health amidst the challenges posed by diabetes and associated conditions..^105^

## 15 Clinical studies that integrated OS in DM with the antioxidants

**Fig. 8:**
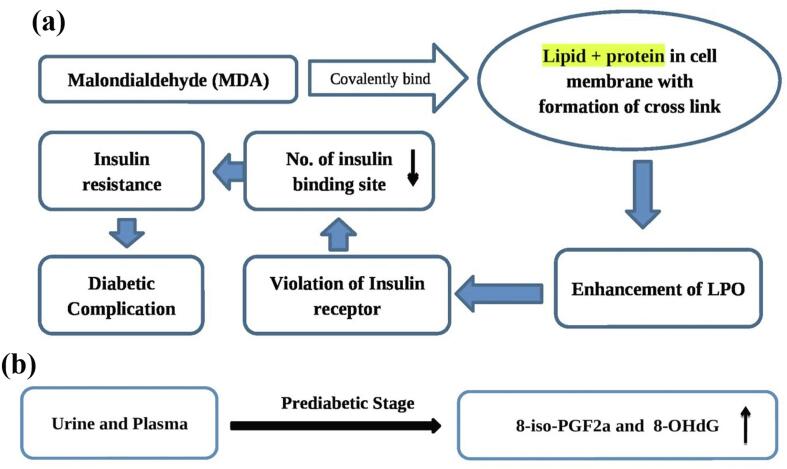
**(a)** Effect of MDA in DM. **(b)** Level of 8-*iso*-PGF2a and 8-OHdG in prediabetic stage.

Most case-control studies suggest that individuals with prediabetes, DM1, and DM2 have higher levels of oxidative damage to lipids, proteins, and nucleic acids than controls. When comparing the results of the patients with DM and micro or macrovascular issues to those without any problems, the results are the same.^92^, ^106^ Therefore, it was observed that in patients with simple DM1, TBA (Thiobarbituric acid)-reactive products were increased and antioxidant enzyme activity was decreased. Malondialdehyde (MDA), concentrations, in the blood of adolescents and children with poor glycemic regulation are much higher than levels that can be controlled and never return to a healthy level. The amount of MDA and the length of DM1 demonstrated a strong relationship between them.^5^ It was found that the increase in LPO reactions alters the interactions between insulin and its particular receptors, due to the capacity of MDA in its ability to covalently attach to proteins and lipids within cell membranes. This results in the formation of cross-linking. This blocks the entry of insulin receptors and reduces the number of sites that are insulin-binding and leads to the progress of insulin resistance.^107^ (Fig. 8**a**).

It was demonstrated that the development of vascular issues within DM1 is correlated with an increase in the AOD deficit as evident from reductions in the number of essential antioxidants (a-tocopherol and ceruloplasmin and glutathione)^108^ (Fig. 8**b**). The amounts of TBA-reactive products, plasma AGE, and urine 8-hydroxy-deoxyguanine (8-OHdG) lesion were the most consistent indicators of OS in individuals with DM2. These markers were strongly associated with glycemia control issues as well as the intensity in the DM2 course. In the majority of these research studies the results showed an increase in 8-is prostaglandin F2a (8-*iso*-PGF2a) and 8-OHdG levels within the urine and in plasma during the prediabetic phase..^109^, ^110^

**Fig. 9:**
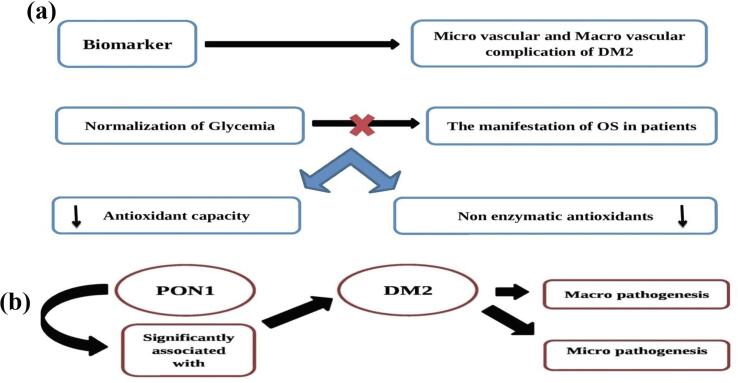
**(a)** Antioxidant capacity of biomarker. **(b)** PON1 in DM2.

The antioxidant system is well-studied for those suffering from DM2 is available. In cases where OS symptoms do not disappear through normalizing anemia, there is a decrease in the total antioxidant capability and non-enzymatic antioxidant levels (like glutathiones) found in the plasma of blood. The treatment with insulin restored OS measurements for DM1 patients but showed no impact on DM2 patients.^111^ (Fig. 9**a**). The results of the study showed an increase in SOD activity, catalase as well as GPx in the red blood cells of people suffering from DM2 and patients with stroke and coronary heart disease. Additionally, In DM patients with a cardiac disease, the SOD GPx and GR activity were drastically reduced and males showed less SOD and GPx activities than women.

**Fig. 10:**
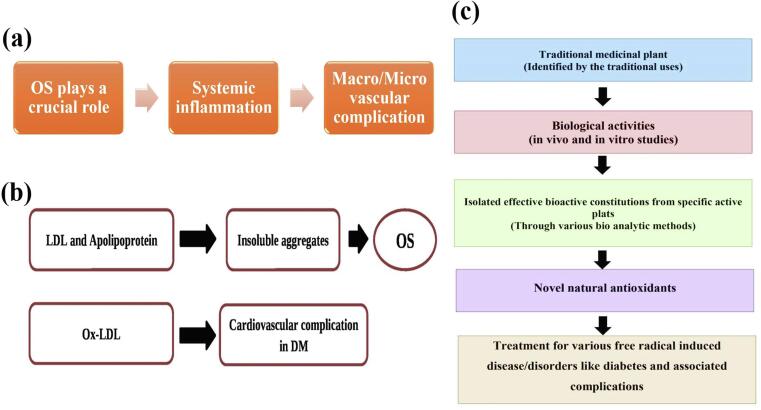
**(a)** OS in vascular complication. **(b)** Relation between OS and LDL, Ox-LDL. **(c)** Natural plant derived products (antioxidants) against different ailments.

The results of a study show a link between paraoxanase one (PON1) and hemoxygenase 1 (HO1) in the blood of those suffering from DM2 and its other consequences. Recent *meta*-analyses have revealed the fact that PON1 plays a significant role in susceptibility to DM2 and the development of micro- and macroangiopathies.^112^ (Fig. 9**b**). The significance of OS in the process of systemic inflammation is clear from the evidence available. It is an important contributor to the pathophysiology that causes macro- and micro-vascular issues in DM patients (Fig. 10**a**). Besides, diabetes dramatically alters the lipid profile of cells and causes them to be more vulnerable to LPO. According to current research, oxidative damage in diabetes complications is caused by LDL lipids and an apolipoprotein component that produces insoluble agglomeration. Based on recent research, the oxidized lipoproteins (Ox-LDL) have a greater impact on those suffering from heart-related complications DM and LDL being oxidized is higher than the control group.^113^ (Fig. 10**b**).

There is no evidence that the frequency of metabolic processes in diabetics is influenced by various aspects, including gender, age, and the influence of ethnicity. A significant variation among ethnicities in diabetes might be due to different habitat conditions (external environmental variables) and the presence and frequency of predisposing and protection genetic markers within diverse groups. Research conducted earlier within the Russian Federation showed a lower incidence of DM in native Northern peoples and people from Siberia, which is explained by the existence of genes that protect that are associated with this nosology.^114^ Therefore, research conducted by researchers from the Research Center for Family Health and Human Reproduction Problems determined that Buryats did not have a very high risk for DM1 and didn't have the same genotypes, or HLA-alleles relationship as Caucasians.^115^ Although the role of many OS biomarkers for the development of vascular complications in diabetes is well-established new methods to assess these markers, specifically the technique of Kinetic Chemiluminescence (determining that the plasma's antioxidant as well as prooxidant levels) are being brought into clinical practice. Its utilization in combination with laboratory, instrumental studies, and clinical trials allows you to assess the health of patients for the selection of diagnosis and treatment.

Redox imbalances can be controlled through an approach specifically tailored to address factors of gender and race *et al*so target ROS sources, which could turn out as an innovative therapeutic strategy for treating diabetes. Herbal Drugs are being used in increasing numbers to treat DM. This is due to their efficacy, reduced side effects, and low costs. These plants contain a variety of active phytoconstituents including alkaloids and glycosides. They also include carbohydrates, polysaccharides (hypoglycans), peptidoglycans (peptidoglycans), guanidines, steroids, glycopeptides, various amino acids, inorganic elements, and terpenoids. These phytoconstituents affect the glucose levels in humans directly and indirectly through their metabolic activity. According to the World Health Organization, approximately 80 percent (or about 80 %) of the global population rely on traditional medicine as their main source of primary healthcare.^116^ (Fig. 10**c**). In general, genetic deletions of antioxidant enzymes increase oxidative stresses and cause insulin resistance or glucose intolerance. Overexpression of antioxidants, however, reduces the oxidative state and improves glucose intolerance and insulin resistance (Table 3)**.**

**Table 3:** Deficiency and excess expression of antioxidant enzymes affect insulin sensitivity, glucose metabolism, and insulin tolerance.^117^, ^118^

Antioxidant	Chemical and Gene modification	Metabolic phenotype
SOD1	Global KO	Muscle mitochondrial production of hydrogen peroxide is increased due to a reduction in the β-cells volume, insulin secretion, and insulin sensitivity.
	Global OE	Enhanced glucose intolerance and decreased oxidative stress and hydrogen peroxide formation in skeletal muscle
SOD2	Hz global KO	Reduced insulin production, elevated reactive oxygen species in islets, and unchanged insulin sensitivity
	Global OE	Enhanced glucose intolerance and decreased oxidative stress and hydrogen peroxide formation in skeletal muscle.
		Maintained insulin sensitivity and decreased production of hydrogen peroxide
	Skeletal muscle OE	Enhanced glucose intolerance and decreased oxidative stress and hydrogen peroxide formation in skeletal muscle.
Catalase	Global KO	Increased oxidative stress and elevated insulin resistance in white adipose tissue.
		Increased oxidative stress and accelerated obesity in white adipose tissue.
	Global OE	Decrease in fat mass, levels of oxidative stress, and glucose.
	Mitochondrial OE	Enhanced glucose intolerance and decreased oxidative stress and hydrogen peroxide formation in skeletal muscle.
		Improved insulin resistance and reduced hydrogen peroxide generation and lipid accumulation in the skeletal muscle.
SOD2 and catalase	Global SOD2 OE and mitochondrial catalase OE	Reduced oxidative stress and hydrogen peroxide production in the skeletal muscles. There is no difference between the mitochondrial catalase-OE and that which only increases hydrogen peroxide production or insulin sensitivity.
GPx1	Global OE	Enhanced fat mass and the development of insulin resistance.
	Global KO	Increased ROS production and improved insulin resistance.
	Liver KO	Hepatocytes with improved insulin sensitivity and increased hydrogen peroxide production.
GPx1 and catalase	Global KO	Prevention of obesity, improved glucose tolerance, and attenuated nonalcoholic fatty liver.
GRx2	Global KO	Increased insulin resistance, obesity, and brain oxidative damage.
Prx2	Global KO	Age-related insulin resistance in muscles is exacerbated by oxidative stress and increased insulin resistance.
		Prevented obesity and insulin resistance.
		No effect on insulin resistance and oxidative damage in the control diet/reduced insulin sensitivity/increased oxidative stresses
Prx3	Global KO	increased amounts of superoxide in 3 T3-L1 adipocytes, enhanced oxidative stress, decreased insulin sensitivity, and impaired glucose tolerance.
	Global OE	Improved glucose tolerance and reduced levels of mitochondrial hydrogen peroxide and oxidative stresses.
MsrA	Global KO	Impaired glucose tolerance and exacerbated insulin resistance and oxidative stress.
	Mitochondrial OE	Improvement of insulin resistance without MsrA cytosolic.
	Cytoplasmic OE	Unaltered insulin resistance.
MsrB1	Global KO	No effect on insulin sensitivity, hydrogen peroxide levels, or oxidative stress.
SelW	Global KO	Enhanced glucose intolerance and decreased oxidative stress and hydrogen peroxide formation in skeletal muscle.

The discovery of free radical scavengers led to a greater awareness regarding the importance of antioxidants for preventing a wide range of human diseases, such as cancer, diabetes, Alzheimer's, and stroke. It is possible that natural antioxidants can be used to treat diabetes, both in terms of preventing its onset and reducing complications.

## 16 Future prospective

In diabetic or insulin-resistant situations, the increased oxidative stress brought on by hyperglycemia may be the cause of the accelerated risk of cardiovascular disease. Evidence from proteomic studies of DM patients also shows activation of oxidative stress pathways and the fact that a combined kidney-pancreas transplant can at least reverse some of these alterations. Vitamin C, Vitamin E, and a-lipoid acids- superoxide-scavengers required for glutathione regeneration. They improve peripheral nerve blood flow, reduce leukocyte adhesion, and prevent cataracts and mesangial growth when administered individually or together. Vitamin C and Vitamin E can also normalize cellular markers such as malondialdehyde and NF-kB..^101^

In healthy individuals, vitamin C treatment restores endothelium-dependent vasodilation that has been compromised by acute hyperglycemia, indicating that hyperglycemia may be a factor in decreased vascular function by producing superoxide anion.^119^ The manganese superoxide dismutase uncouples oxidative phosphorylation and is an inhibitor of electron transfer chain complex II. The normalization of the levels of mitochondrial oxygen radicals by these agents inhibits glucose-induced activation and activation of Protein Kinase C, accumulation of sorbitol, and activation of NFkB.^72^ The use of SOD/catalase mimics in subjects suffering from DM appears promising. The mimetics could act by decreasing the superoxide excess that inhibits antiatherosclerotic enzymes such as eNOS and prostacyclin synthase. Treatment with SOD/catalase mimics in diabetic mice prevents the oxidative inhibition of aortic procyclin synthase induced by diabetes and normalizes different pathways involved in hyperglycemic injury. The diacylglycerol (DAG)-protein kinase C (PKC) pathway, the AGE production pathway, and the hexosamine pathway are three of the main metabolic processes linked to the pathophysiology of hyperglycemia-induced vascular injury. Benfotiamine, a lipid-soluble thiamine derivative, can block both of these routes and the NF-kB activation linked to hyperglycemia. Benfotiamine has been shown to counteract glucose-mediated toxicity, in both mouse and cultured cell models. The drug also shows beneficial effects in the pathways that are associated with diabetic complications..^103^

## 17 Conclusion

As oxidative stress is a lack of balance between the pro-oxidant and the antioxidant species which is a key factor in diabetes complications that lead to microvascular problems. Therefore, the overview on the DM-OS metabolic effects relationships with antioxidant mechanisms could enhance the crucial next step in the ROS inclusion within sustainable models. Conventional studies on the antioxidants, such as vitamin E, are still trying to prove the exact mechanism of action that could link the DM-OS with the treatment of humans CVD through their high potentials on neutralizing the oxygen-reactive molecules and superoxide metabolites. In which, they could significantly enhance the antioxidant enzymes catalytic actions, such as SOD/catalase that could decrease CVD consequences. Indeed, reducing OS leads to a higher survival rate and a reduction in diabetes co-morbidities. Where, research in the future should concentrate on the possibility of determining the benefits of fixed-combination therapies in decreasing oxidative stress, and also identifying drugs or food components which may have a positive effect on OS. Besides, establishing of molecular fingerprints for the antioxidants bioactive components based on their therapeutic properties and mechanism of actions will help the research community to make further insights that could face the DM global burden complications.

## Funding

This work was funded by National Natural Science Foundation of China (Project No: 32171889), the “Belt and Road” joint project fund between Zhejiang University, China, and the National Research Centre, Egypt (Project No: SQ2023YFE0103360), and the Key R&D Projects in Zhejiang Province (Project No: 2023C02009).Also, Date Palm Research Center of Excellence (DPRC), King Faisal University (KFU), Saudi Arabia, funded the current study (Project No: DPRC-10-2024)..

## CRediT authorship contribution statement

**Shouvik Mallik:** Writing – review & editing, Writing – original draft, Investigation, Conceptualization. **Bijoy Paria:** Writing – original draft, Investigation, Data curation. **Sayed Mohammad Firdous:** Writing – review & editing, Writing – original draft, Data curation, Conceptualization. **Hesham S. Ghazzawy:** Investigation, Funding acquisition. **Nashi Alqahtani:** Funding acquisition, Investigation. **Yong He:** Investigation, Resources, Writing – review & editing. **Xiaoli Li:** Data curation, Methodology, Resources, Writing – review & editing. **Mostafa M. Gouda:** Writing – review & editing, Writing – original draft, Visualization, Validation, Supervision, Investigation, Data curation, Conceptualization.

## Declaration of competing interest

The authors declare that they have no known competing financial interests or personal relationships that could have appeared to influence the work reported in this paper.
